# A structural view of ligand-dependent activation in thermoTRP channels

**DOI:** 10.3389/fphys.2014.00171

**Published:** 2014-05-05

**Authors:** Ximena Steinberg, Carolyne Lespay-Rebolledo, Sebastian Brauchi

**Affiliations:** ^1^Faculty of Medicine, Institute of Physiology, Universidad Austral de ChileCampus Isla Teja, Valdivia, Chile; ^2^Faculty of Sciences, Graduate School, Universidad Austral de ChileCampus Isla Teja, Valdivia, Chile; ^3^Faculty of Chemical and Pharmaceutical Sciences, Graduate School, Universidad de ChileSantiago, Chile

**Keywords:** TRP channels, TRPV1, TRPM8, structure, PIP_2_, capsaicin, menthol

## Abstract

Transient Receptor Potential (TRP) proteins are a large family of ion channels, grouped into seven sub-families. Although great advances have been made regarding the activation and modulation of TRP channel activity, detailed molecular mechanisms governing TRP channel gating are still needed. Sensitive to electric, chemical, mechanical, and thermal cues, TRP channels are tightly associated with the detection and integration of sensory input, emerging as a model to study the polymodal activation of ion channel proteins. Among TRP channels, the temperature-activated kind constitute a subgroup by itself, formed by *Vanilloid* receptors 1–4, *Melastatin* receptors 2, 4, 5, and 8, TRPC5, and TRPA1. Some of the so-called “thermoTRP” channels participate in the detection of noxious stimuli making them an interesting pharmacological target for the treatment of pain. However, the poor specificity of the compounds available in the market represents an important obstacle to overcome. Understanding the molecular mechanics underlying ligand-dependent modulation of TRP channels may help with the rational design of novel synthetic analgesics. The present review focuses on the structural basis of ligand-dependent activation of TRPV1 and TRPM8 channels. Special attention is drawn to the dissection of ligand-binding sites within TRPV1, PIP_2_-dependent modulation of TRP channels, and the structure of natural and synthetic ligands.

## TRP channels

Mammalian TRP channel proteins are polymodal cation channels that participate in sensory physiology at different levels. This includes thermo-sensation, mechano-sensation, nociception, touch, taste, olfaction, and vision (Clapham, 2003; Voets et al., 2005). Other than a loose sequence homology, predicted channel architecture, and non-selective cation permeability, there are no particular features defining the TRP family. The 28 known mammalian TRP channels are grouped by homology into six sub-families named C, M, V, A, P, and ML, for canonical, melastatin related, vanilloid binding, ankyrin repeat, polycystin, and mucolipin, respectively, (Ramsey et al., 2006). By integrating multiple stimuli they supply signal amplification through calcium permeation and membrane depolarization. Cooperativity intrinsic to TRP channels may result in allosteric coupling of distinct activation stimuli. A good example of the allosteric nature of TRP channels would be the case of temperature-activated TRP channels (thermoTRPs) (Latorre et al., 2007; Pertusa et al., 2012).

Together with their role in sensory and pain physiology (Cesare and McNaughton, 1996; Caterina et al., 1997; Tominaga et al., 1998; De Petrocellis et al., 2008; Julius, 2013) TRP channel function is underscored by the prevalence of various diseases, for example hypomagnesemia, polycystic kidney disease, and mucolipidosis, caused by mutation of TRP proteins (Montell, 2005; Nilius, 2007). All this is accompanied by recent advances on menthol-based cancer treatments (Journigan and Zaveri, 2013). Consequentially, the role of TRP channels in the regulation of several sensitive processes as well as in physiopathology has led to the identification of a diverse set of ligands with potential therapeutic applications (Messeguer et al., 2006; Nilius, 2007; Latorre et al., 2009; Alawi and Keeble, 2010).

## General architecture of temperature-activated TRP channels

After the cloning of the capsaicin receptor TRPV1 (initially dubbed VR1) by the group of David Julius (Caterina et al., 1997), several TRP channels have been identified as thermal sensors. To date, temperature-activated TRP channels correspond to a subgroup of 10 TRP channels which are expressed in sensory nerve endings and skin cells, and are characterized by distinctive high temperature-dependent activation (Q_10_ > 10). ThermoTRP channels display different dynamic ranges for their temperature activation profile (Pertusa et al., 2012), are activated by several natural compounds (Hwang et al., 2000; Palazzo et al., 2013; Ramachandran et al., 2013), and are also identified through their participation in nociceptive pathways (Cesare and McNaughton, 1996; Caterina and Julius, 1999; Di Marzo et al., 2002; Immke and Gavva, 2006; Mandadi and Roufogalis, 2008; Dux et al., 2012; Straub et al., 2013).

One interesting feature of thermoTRP channels is the presence of members from at least four different TRP families (V, M, A, and C). Whereas TRPV1–4, TRPM2, TRPM4, and TRPM5 are heat-activated, TRPM8, TRPA1, and TRPC5 are activated by cold (Patapoutian, 2005; Clapham, 2009; Zimmermann et al., 2011; Pertusa et al., 2012). We focus our attention on TRPV1 and TRPM8, the most studied thermal receptors for hot- and cold-sensing, respectively (Latorre et al., 2009).

**Figure 1:**
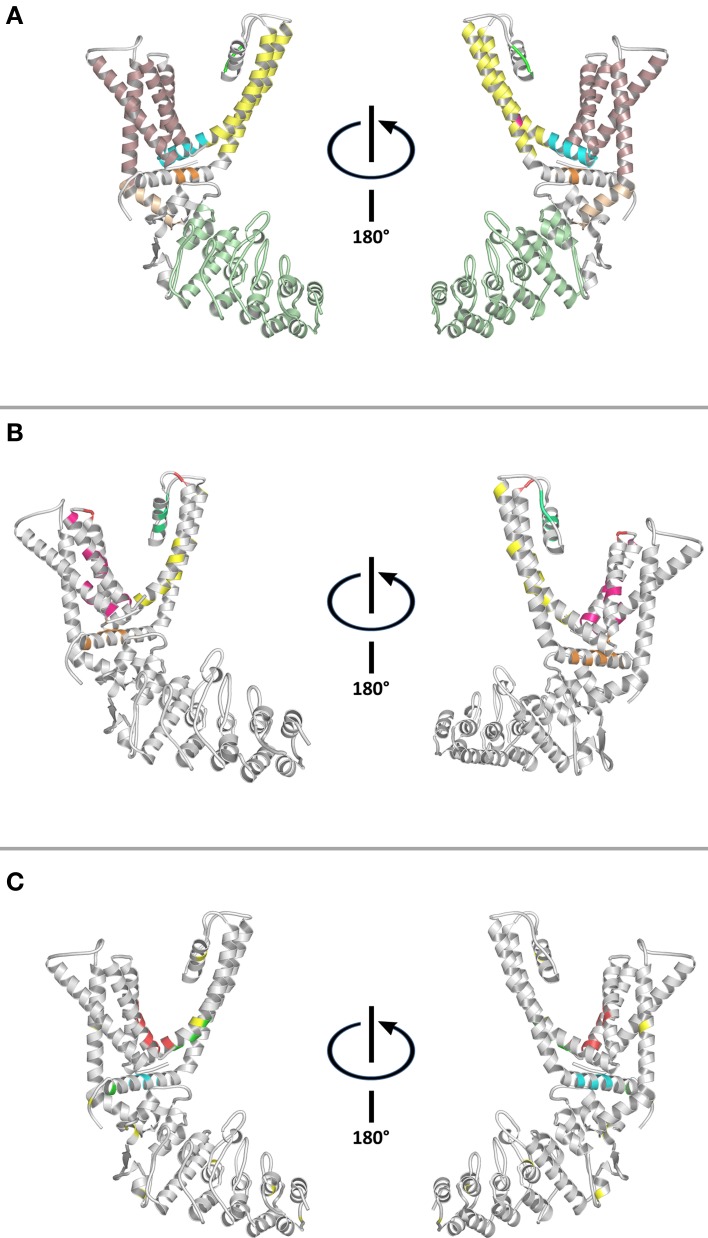
**Structural features of the capsaicin receptor. (A)** Conserved structural domains: Ankyrin repeat domain—Olive. Pre-TM1 helix—Salmon. TM1-TM4 domain—Pale pink. TM4-TM5 linker—Cyan. Selectivity filter—Green. Gate—Fuchsia. TM5-TM6 domain—Yellow. TRP domain—Orange. **(B)** Residues involved in ligand-binding and/or modulation of channel activity: Colors represent residues location. TM1-TM4 domain—Fuchsia. Selectivity filter and pore helix—Green. TM5-TM6 domain—Yellow. Intracellular loops—Orange. Extracellular loops—Red. **(C)** Putative ligand-binding sites: Vanilloids—Red. Fatty acids and lipids—Green. PIP_2_—Cyan. Cysteine residues—Yellow. TRPV1 structure (PDB ID 3J5P) was visualized and colored using PyMOL Molecular Graphics System.

Phylogenetic studies, transmembrane segment prediction, and structural data indicates that TRP channels are related to the superfamily of voltage-gated cation channels, for example voltage-gated potassium (K_v_) and voltage-gated calcium (Ca_v_) channels (Phillips et al., 1992; Wes et al., 1995; Harteneck et al., 2000; Ramsey et al., 2006; Liao et al., 2013). Biochemical, optical, and structural information support the notion of a tetrameric channel architecture (Jahnel et al., 2001; Kedei, 2001; Amiri et al., 2003; Veliz et al., 2010; Liao et al., 2013). TRP channels are diverse in their design and numerous structural features vary between members of different sub-families. However, the functional TRP channel is formed by the coassembly of four subunits, each comprising six transmembrane-spanning (TM) helices with intracellular amino- and carboxyl-terminus domains (Harteneck et al., 2000). By analogy to the 6TM architecture of K_v_ channels, TM5 and TM6 constitute the pore region. Electrophysiological and structural data further support this topology (Owsianik et al., 2006; Susankova et al., 2007; Salazar et al., 2009; Liao et al., 2013; Figure 1A). Since TRP channels are considered distant relatives of K_v_ channels and elicit voltage-dependent activation (*zd* = 0.4e − 0.9e), the TM1-TM4 region has been suggested to serve as a voltage-sensing domain (VSD) (Voets et al., 2007; Boukalova et al., 2013). However, chimeras between TRPM8, TRPV1, and K_v_1.2 in which TM1-TM4 of TRPM8, and TRPV1 is replaced with TM1-TM4 of K_v_1.2 produced non-functional TRP channels suggesting that the K_v_1.2 VSD is insufficient to restore TRP channel function (Kalia and Swartz, 2013). The structure of TRPV1 lacks a patch of charged amino acids, located in the TM1-TM4 domain, typically associated with voltage-sensitivity in K_v_ channels. Thus, evidence indicates that TRP channels likely utilize a different mechanism to sense voltage. Given a hypothetical scenario in which the TM1-TM4 domain behaves statically, as it apparently does when ligands or toxins bind to the channel (Cao et al., 2013b), it is probably not TM1-TM4 but the pore region undergoing voltage-dependent structural rearrangements. It is important to note here that most of the work claiming voltage-dependent changes caused by mutagenesis underscore a shift in the conductance-voltage (G-V) curve along the voltage axis as an indication of voltage dependence. This should be taken with caution since this observation alone may suggest a direct effect on allosteric coupling rather than gating charge suppression.

Alanine-scanning mutagenesis of the TRPV1 pore domain identified three residues critically involved in capsaicin, thermal and pH activation: Y671, I672, and N676 (Susankova et al., 2007). Interestingly, the mutation Y671A dramatically reduces capsaicin TRPV1 response, and the characteristic heat potentiated response to agonist is abolished. Promotion of agonist desensitization when repeated capsaicin pulses were applied caused heat-induced potentiation recovery. The authors hypothesized that Y671 may be involved in the allosteric mechanism coupling thermal and agonist activation (Susankova et al., 2007). This observation was particularly interesting since SCAM analysis (Substituted Cysteine Accessibility Method) performed by the Rosenbaum group (Salazar et al., 2009) locates that residue in the narrowest region of the pore. The recent cryo-electron microscope (cryo-EM) structure of TRPV1 (Cao et al., 2013b; Liao et al., 2013) shows a constriction point at position Y671, but suggests that the narrowest constriction lies right below, at residue I679.

In addition, it has been suggested that packaging and coupling of the TM1-TM4 module differs considerably between K_v_1.2 and TRP channels (Kalia and Swartz, 2013). This is in good agreement with fluorescence spectrometry functional experiments in which the authors measured a set of intermolecular distances between C- and N-terminal and with respect to the plasma membrane, and fitted those to the low resolution cryo-EM TRPV1 structure (Moiseenkova-Bell et al., 2008; De-la-Rosa et al., 2013). The results suggest that the molecular packaging of TRPV1 should be more constrained than other voltage-dependent channels. The recent 3.4 Å resolution structure of TRPV1 confirms these functional experimental-based predictions; comparison of unliganded (apo) and ligand-bound complexes suggests that the TM1-TM4 domain acts as a rigid body during activation (Cao et al., 2013b).

The TM1-TM4 domain has been mapped to contain most of the ligand binding-related residues (Winter et al., 2013), emerging as a *ligand-binding domain* (LBD) for TRP channels (Figure 1). Furthermore, sensitivity to ligands is maintained by transferring TM3-TM4 moieties of TRPM8, and TRPV1 onto K_v_1.2 (Kalia and Swartz, 2013). TRPV1's structure reveals that the ligand-binding pocket is located near the TM3-TM4 transmembrane region, and the TM4-TM5 linker (Figures 1B,C). Closer inspection of the reported structures suggest that intra- and inter subunit contacts between the ankyrin repeat domain (ARD)-TM1 linker, TM4-TM5 linker, and proximal C- terminal region might be modified in response to agonists.

## Molecular modeling of TRP channels

Structures belonging to the superfamily of potassium channels, for example *Shaker*-like K_v_1.2 (Long et al., 2005; Chen et al., 2010), cyclic nucleotide-gated (CNG) (Clayton et al., 2008), and hyperpolarization-activated (HCN) (Zagotta et al., 2003) channels have been used as templates to make homology models of the full transmembrane domain of TRPV1 including N-terminal ankyrin repeats, and the C-terminal TRP domain (Table 1). The first structural data on TRPV1 came from cryo-EM, giving support to the generalities accounted for by researchers when modeling TRPs (Moiseenkova-Bell et al., 2008). The evident similarities between TRPV1's low resolution cryo-EM and the structural arrangement of K_v_1.2 is now reinforced by the recent higher resolution cryo-EM data (Liao et al., 2013). It is very important to keep in mind the fact that cryo-EM require the acquisition of multiple pictures of the electronic density of protein's surface, to finally build enriched electron density maps, allowing the 3D reconstruction of the molecule. Therefore, the exact position of the side chains are not resolved in detail, and must be modeled instead of being fitted with precision as is achievable with X-ray crystallography. In this sense, the novel structure can be considered as a well refined model obtained under strict constrains. Nevertheless, the novel TRPV1 cryo-EM structures provide valuable information about the tetrameric architecture and ligand-binding regions, promoting the re-evaluation of current models and their predictions (Cao et al., 2013b; Liao et al., 2013; Table 1).

**Table 1:** **Templates used for homology modeling of TRPs and the packing of LBD of TRPV1 and TRPM8 channels**.

**Protein**	**Method used**	**Structure or structural template with PDB identifier**		**Packing of LBD**	**References**
TRPV1	Homology modeling	- S1–S6	2R9R (Kv1.2)	n/d	Brauchi et al., 2007
		- C- termini	1Q3E (HCN2)		
TRPV1	Homology modeling	- N-termini (TMP kinase)	1E9C	Loose	Fernández-Ballester and Ferrer-Montiel, 2008
		- Ankyrin domain (TRPV1 ARD)	2PNN		
		- S1–S6 (Kv1.2)	2R9R		
		- Proximal C-termini (synthetic)	1WL5		
		- Distal C- termini1 (HCN2 channel)	Q3E		
TRPV1	CryoEM	- Ankyrin domain (TRPV1 ARD)	2PNN	n/d	Moiseenkova-Bell et al., 2008
		- S1–S6 (Kv1.2)	2R9R		
TRPM8	Homology modeling	13 different protein fragments. Including:		Loose	Pedretti et al., 2009
		- S3 and S4 (Kv1.2)	2R9R		
		- C- termini (HCN2-cAMP)	1Q50		
TRPM8	TRPM8 and TRPM8-ago nist MD simulations	TRPV1 homology model by Pedretti et al. (2009).		Loose	Pedretti et al., 2011
TRPV1	Fluorescence spectroscopy and CryoEM	- Ankyrin domain	2PNN	Constrained	De-la-Rosa et al., 2013
		- S1–S6	2R9R		
TRPM8 TRPV1	Homology modeling and further testing by chimeric constructs	Independently:		Constrained	Kalia and Swartz, 2013
		(Kv1.2)	2R9R		
		(MLotiK)	3CL1		
TRPV1	CryoEM for apo TRPV1 channel	2R9P		–	Liao et al., 2013
TRPV1	CryoEM for RTX/DkTx bonded TRPV1 channel	2R9Q		–	Cao et al., 2013b
TRPV1	CryoEM for capsaicin bonded TRPV1 channel	2R9R		–	Cao et al., 2013b

n/d, not described.

An extensive overview on the structure of TRP channels fragments with high resolution data can be found in “Structural Biology of TRP channels,” by Li et al. (2011).

Molecular modeling is an extremely useful methodology for complementing experimental techniques as it provides a potential structural context for interpreting experimental results. The high sequence homology among TRPVs will allow researchers to construct molecular models of other members of the TRPV sub-family and help bridge the structure-function paradigm. Furthermore, the promiscuous behavior among TRP channels toward ligands suggests a conserved LBD and lends support to future molecular models of TRP channels. However, molecular models for TRP channels members of other sub-families should be constructed with extreme caution because low resolution EM structures obtained for other TRP channels such as TRPC3 and TRPM2, present a different three dimensional arrangement, looking awkwardly bulky (Maruyama et al., 2007; Mio et al., 2007).

## The TRP domain and PIP_2_-dependent modulation of thermoTRP channels

Ligands of lipidic nature act as endogenous regulators of thermoTRP channel activity (Lukacs et al., 2013; Poveda et al., 2013). For the case of TRPV1, this includes negatively charged lipids such as phosphatidylinositol 4,5-bisphosphate [PIP_2_, PI(4,5)P_2_], lysophosphatidic acid (LPA), and *N*-arachidonoyl-dopamine (NADA) (well reviewed by Morales-Lázaro et al., 2013). As one of the most important endogenous ligands, we focus our attention on the PIP_2_-dependent modulation of channel activity and structural features of the TRP domain.

The TRP domain, a 25 amino acid sequence located immediately after the TM6 transmembrane helix is a shared feature among channels from the C, V, and M subfamilies. This domain contains the TRP box, a highly conserved region originally dubbed the “*EWKFAR motif*” (Zhu et al., 1995). The TRP box is defined by the consensus sequence WKFQR, in which tryptophan is the most conserved residue. The positive charges at positions 2 and 5 of the sequence are shared by almost all TRPs having a TRP domain including TRPV1 and TRPM8 channels, and have been suggested as part of the PIP_2_ sensor of the channel (Latorre et al., 2009).

PIP_2_ is an abundant, short-lived phospholipid present in the plasma membrane of cells (Le Roy and Wrana, 2005), playing a central role in regulating ion channel activity (Kruse et al., 2012). It has been shown that most TRP channels from the C, M, and V families are regulated by PIP_2_ (Nilius et al., 2008; Rohacs, 2009; Morales-Lázaro et al., 2013). The hypothesis is that positively charged residues present in the TRP domain are responsible for PIP_2_-dependent modulation of TRP channel activity (Rohacs et al., 2008; Latorre et al., 2009) sensitizing TRPV and TRPM channels (Ferrer-Montiel et al., 2004; Rohács et al., 2005; Brauchi et al., 2007; Rohacs et al., 2008; Stein et al., 2009; Ufret-Vincenty et al., 2011; Fujita et al., 2013). Recently by biochemistry, electrophysiology, and FRET imaging, a positively charged amino-terminal sequence has been identified in TRPV4, and proposed as PIP_2_ binding pocket (Garcia-Elias et al., 2013). Thus, as suggested for other TRPs, upon PIP_2_ interaction a reconfiguration favoring channel activation occurs. Of worthy note is the fact that the TRP domain may be bimodal; for example Doerner et al. clearly demonstrate that TRPV3 channels are sensitized by depletion of PIP_2_ in a TRP domain-specific fashion (Doerner et al., 2011). Additionally, the work of David Julius shows a desensitizing effect of PIP_2_ over TRPV1 activation (Prescott and Julius, 2003; Cao et al., 2013a). Such an effect, demonstrated on intact cells and liposome reconstituted channels, is thought to be mediated by the distal portion of the C-terminal domain, absent in the cryo-EM structure of the channel. Thus, it is still an interesting matter of discussion how PIP_2_ binding promotes potentiation or reduction of ionic currents derived from thermal, agonist, or voltage activation.

**Figure 2:**
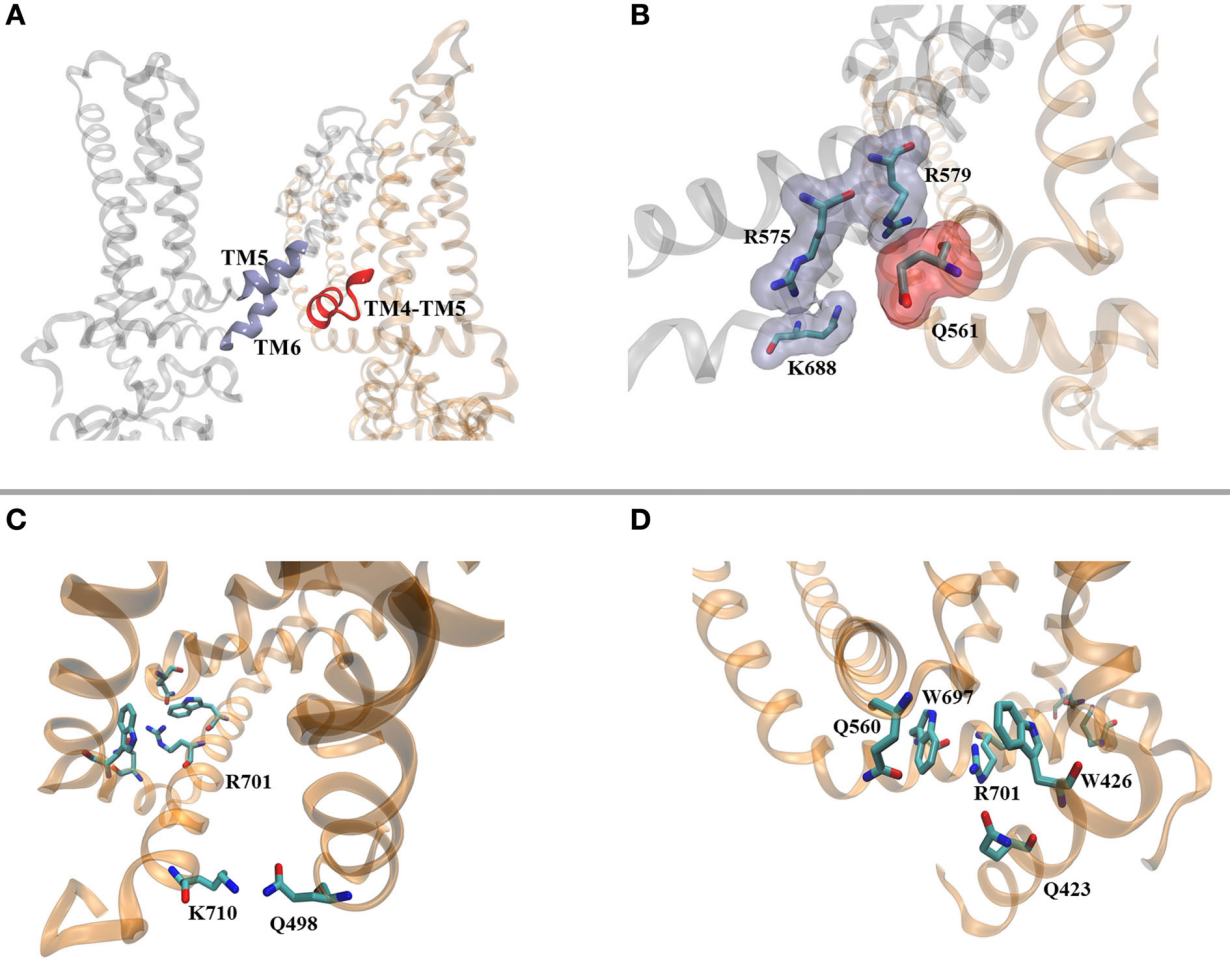
**PIP_2_ mediates intra- and inter-subunit contacts. (A)** Identified PIP_2_ binding pocket in the structure of TRPV1. **(B)** Residues proposed as mediators of PIP_2_ interaction to the channel. **(C)** K710 is located at the distal end of the TD helix and interacts with Q498 located at the bottom of TM2. **(D)** Predicted/potential cation-π interactions connecting the TRP box of the TD helix with the TM4-TM5 helix and N-terminal region. In order to get a better placement of the lateral chains of the available structure, the TRPV1 channel (PDB ID 3J5P) was embedded in lipids (POPC), and placed in a periodic box containing water and ions (140 mM KCl). After 5 ns of all-atom MD simulations using the NAMD/CHARMM32 force-field, the channel was visualized using VMD.

It has been proposed that PIP_2_ determines intra- and inter-molecular connectivity, controlling allosteric coupling within TRP channels (Brauchi et al., 2007; Garcia-Sanz et al., 2007; Latorre et al., 2009; Figure 2). The availability of a cryo-EM TRPV1 structure in its apo, closed state, and 3D reconstructions of activated states in complex with the vanilloid agonists resiniferatoxin (RTX), and capsaicin permits comparison of different functional states of TRPV1 (Cao et al., 2013b). This approach is likely to enhance understanding of the activation of TRP channels by different agonists and identify potential gating sites. Inspection of apo and ligand-bound structures of TRPV1 (Cao et al., 2013b; Liao et al., 2013; PDB IDs: 3J5P, 3J5Q, and 3J5R), identified a binding pocket suitable to fit one PIP_2_ molecule connecting TM5 and TM6 from one subunit with the TM5-TM6 linker of the neighbor subunit (Figure 2A). This binding pocket is similar to the one predicted earlier by molecular docking and molecular dynamics (MD) simulations (Brauchi et al., 2007), and is in good agreement with the position of PIP_2_ molecules in the crystal structures of inwardly rectifying potassium channels Kir2.2 and Kir3.2 (Hansen et al., 2011; Whorton and MacKinnon, 2011; PDB IDs: 3SPI, and 3SYA).

In the case of Kir channels, the phosphoinositide is coordinated by residues located on the lower part of the outer helix and tether helix, establishing inter-molecular interactions through bonding to the IF-helix of the neighboring subunit (see note below^1^) Our previous work (Brauchi et al., 2007), and preliminary MD simulations on the recent structural data shows that TRPV1's putative binding pocket for PIP_2_ elicit a patch of four charged residues (Q561, R575, R579, and K688), readily available for the coordination of the negatively charged head group of the lipid (Figures 2A,B). Interestingly, as in Kir channels, these residues are located in different subunits making possible the existence of a bridge between them as we envisioned before (Brauchi et al., 2007; Latorre et al., 2009). In support of this hypothesis, mutational studies within this region identified amino acids producing strong desensitization of the channel (Q560, Q561, R575, R579, K688, W697, R701, and K710; Winter et al., 2013). According to these results, R701 and K710 located in the TRP domain are also predicted as PIP_2_ binding residues. However, according to our view, it seems that there is little space for them to interact with the polar head group of the lipid.

The three dimensional arrangement of the TRP domain helix (TD helix) in the context of the TRPV1 structure allows us to draw an alternative hypothesis. The structure shows the location of the TRP box defined by the sequence ^697^WKLQR^701^ at the central portion of the TD helix, followed by the sequence ^702^AITILDTEK^710^ on the second half (Figure 2C). The charged residues R701 and K710 have been suggested as negatively charged residues coordinating PIP_2_, however they appear closer to the aromatic residues W697 (TD helix) and W426 (N-terminal). This is a suitable situation for the formation of cation-π interactions controlling TRPV1's gate (Figure 2C) and represent an interesting structural feature to explore. Recent reports from the group of A. Ferrer-Montiel (Valente et al., 2008; Gregorio-Teruel et al., 2014) showed that amino acids I696 and W697, located within the TD helix, are essential for channel's function, probably by controlling the correct allosteric coupling of the ligand-dependent conformational change and voltage-sensor movement to the channel's gate, suggesting that they are part of a more complex intra- or inter-molecular network of connectivity. The TD helix is positioned horizontal to the plane of the membrane. In order to promote and/or control channel gating we envisioned a sliding movement of the TD helix either linear, or lateral with a pivot joint around K710. This sliding helix would be controlled by interactions associated to W697, acting as a latch. More than half of the cation-π pairs involve arginine residues and about one-third asparagine or glutamine residues carrying a partial charge (Dougherty, 1996). Although the resolution of the cryo-EM structure is insufficient to accurately place the side chains rotamers, it is tempting to suggest that the hypothetical cation-π core identified in this region may be helped by the presence of residues Q560 (TM4-TM5 linker) and Q423 (N-terminus). The residues Q560 and Q423 (together with W426), are located at the bottom of the ligand binding pocket, and close to a region that has been associated with thermal sensitivity, respectively (Yao et al., 2011), interestingly, the modification of any of these residues induce strong desensitization of the channel (Winter et al., 2013). Thus, we envision a cluster of cation-π interactions as the molecular switch connecting different stimuli to the gate. At the C-terminal end of the TD helix K710 appears to be in close proximity to Q498, located in the TM2-TM3 linker (Figure 2D). It is possible that upon PIP_2_ binding to the proximal TD helix the interactions observed in the TRP box will be re-shaped, unlocking the TD helix leading to channel opening. Such negative control of the gate by the TD helix has been suggested previously (Garcia-Sanz et al., 2007). Further studies addressing the details defining the mechanics of this fundamental region are required for a deeper understanding of TRP channel gating.

## A ligand binding domain

Capsaicin, a pungent ingredient of chili peppers, is *the* natural agonist of TRPV1 channels and is responsible for the spiciness of the plant's fruit. Essential research using capsaicin-insensitive animal models, site-directed mutagenesis, and structural data pinpoint residues located in the TM4-TM5 linker, and TM3 helical residues Y511 and S512 which may stabilize the binding of capsaicin via polar interactions and hydrophobic contacts, respectively (Jordt and Julius, 2002; Cao et al., 2013b). Residues M547 and T550 in TM4 have also been mentioned as part of the vanilloid binding mechanism (Gavva et al., 2004; Cao et al., 2013b). Cao and colleagues suggested that upon capsaicin (or the natural vanilloid resiniferatoxin, RTX) binding to the TM3-TM4 helix and TM4-TM5 linker, hydrogen interactions between the linker and the TM6 helix terminal region are disrupted, presumably promoting gate opening. When comparing TRPV1 ligand-bound cryo-EM structures, it could be observed that capsaicin binding led to gate opening without major changes in selectivity filter conformation and diameter. In contrast, resineferatoxin (RTX/DkTx) binding induced visible alterations within the selectivity filter leading to a wider open pore conformation, somewhat supporting the dilated pore phenotype observed previously (Chung et al., 2008; Cao et al., 2013b). This evidence also suggests the existence of different activation pathways for the agonists, despite the close proximity of their respective binding pockets. Capsazepin (CPZ) is a potent TRPV1 antagonist and in the same way it is observed for capsaicin, residues I514 and V518 from TM3, M547 from TM4, as well as residues located on TM2-TM3 linker are candidates to line the CPZ binding pocket (Phillips, 2004). Following the same trend, several residues in TM2 of TRPM8 have been implicated in menthol- (Bandell et al., 2006) and icilin-dependent activation (Chuang et al., 2004). Amino acids within or near the intracellular TM2-TM3 linker have also been suggested as ligand-binding residues (Voets et al., 2007). Bandell and colleagues have performed a high throughput mutagenesis analysis on TRPM8 channels (Bandell et al., 2006). One interesting outcome is that residues from the TM2 helix were only participating in menthol- and icilin-dependent activation, dissecting the ligand activation mechanism from the other sensory cues (i.e., temperature or voltage). The analogy with TRPV1 is evident, suggesting that the main function of the TM1-TM4 region is to serve as a LBD conserved among thermoTRP channels.

## Vanilloids and methylamine derivatives

The identification and synthesis of derivatives with different chemical modifications have contributed to obtaining a pattern of structure-function relationships, allowing the elucidation of the chemical environment mediating the interactions of the ligands with their target residues in the receptor. Such methodologies ultimately allow the determination of specific shapes and electrostatic factors that contribute to ligand sensitivity, selectivity, and potency. In the next section we discuss some of the properties of thermoTRP channel ligands.

**Figure 3:**
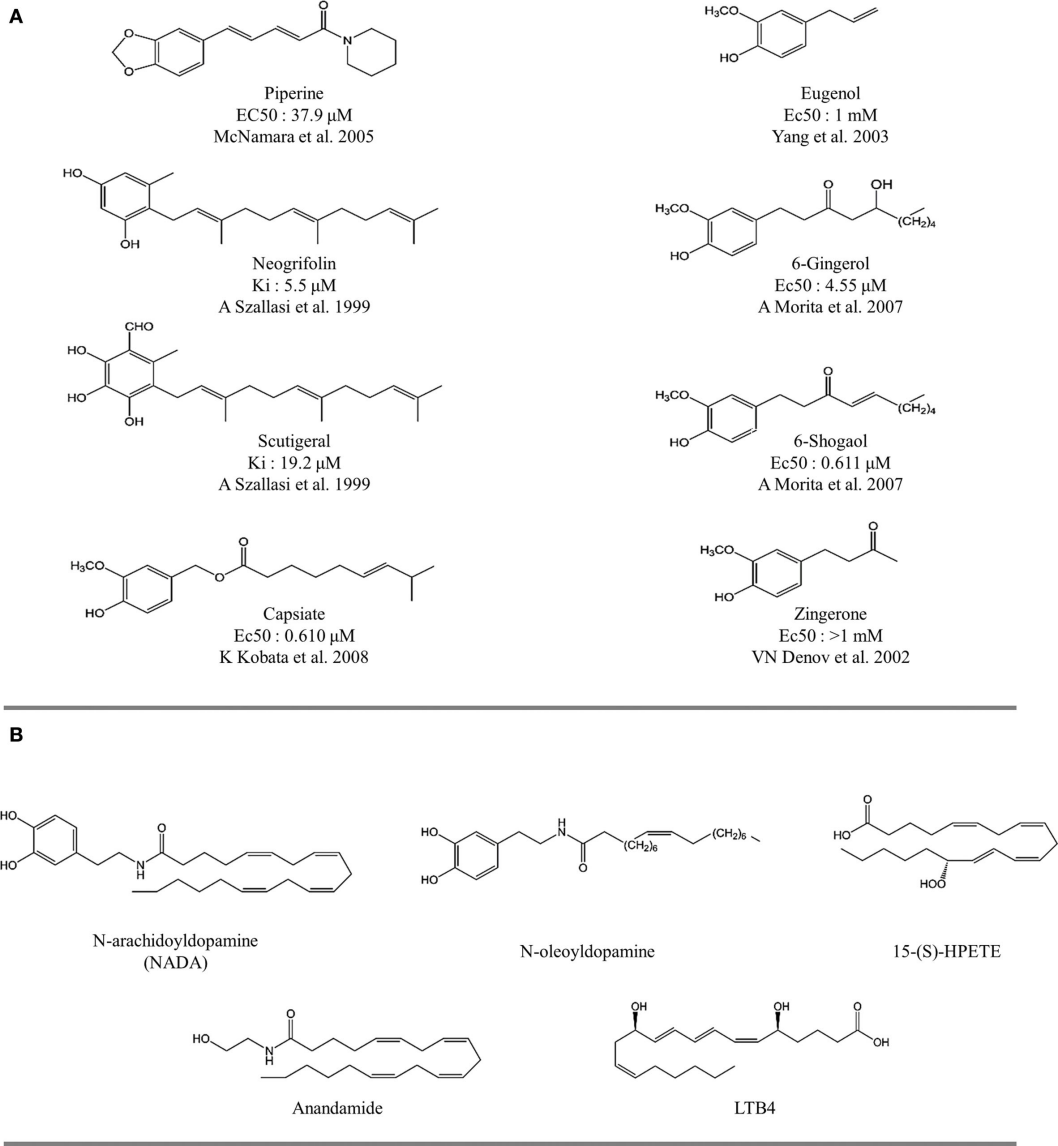
**The structure of natural and endovaniloids. (A)** Natural compounds with agonist activity on TRPV1 receptors. Figure indicates the affinity for specific ^3^[H]-resiniferatoxin binding sites in rat spinal cord (Szallasi and Blumberg, 1999). **(B)** Structure of endovanilloids identified as activators of TRPV1 receptors.

**Figure 4:**
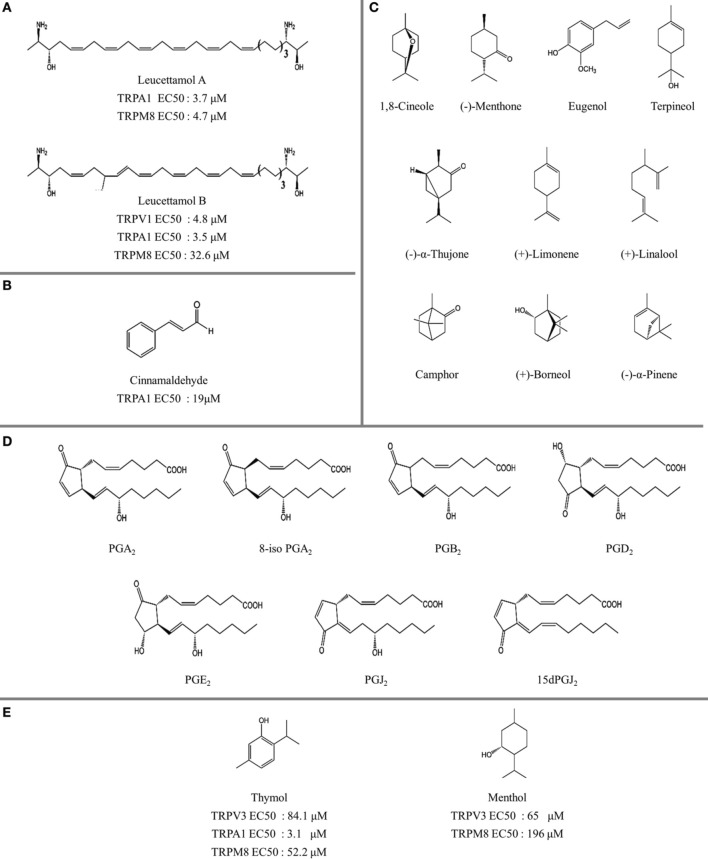
**The structure of natural thermoTRP ligands. (A)** Leucettamol A and B identified with TRP channel activity (Chianese et al., 2012). **(B)** Cinnamaldehyde, TRPA1 activator (Macpherson et al., 2007). **(C)** Structure of monoterpenoids and related compounds evaluated on TRPM8, TRPV3, and TRPA1 receptors (Vogt-Eisele et al., 2007; Takaishi et al, 2012). **(D)** Structure of prostaglandins evaluated in TRPA1 receptors (Taylor-Clark et al., 2008). **(E)** Activity profile of human TRPA1 and mouse TRPM8 channels after addition of thymol (Lee et al., 2008) and menthol (Sherkheli et al., 2010), respectively.

**Figure 5:**
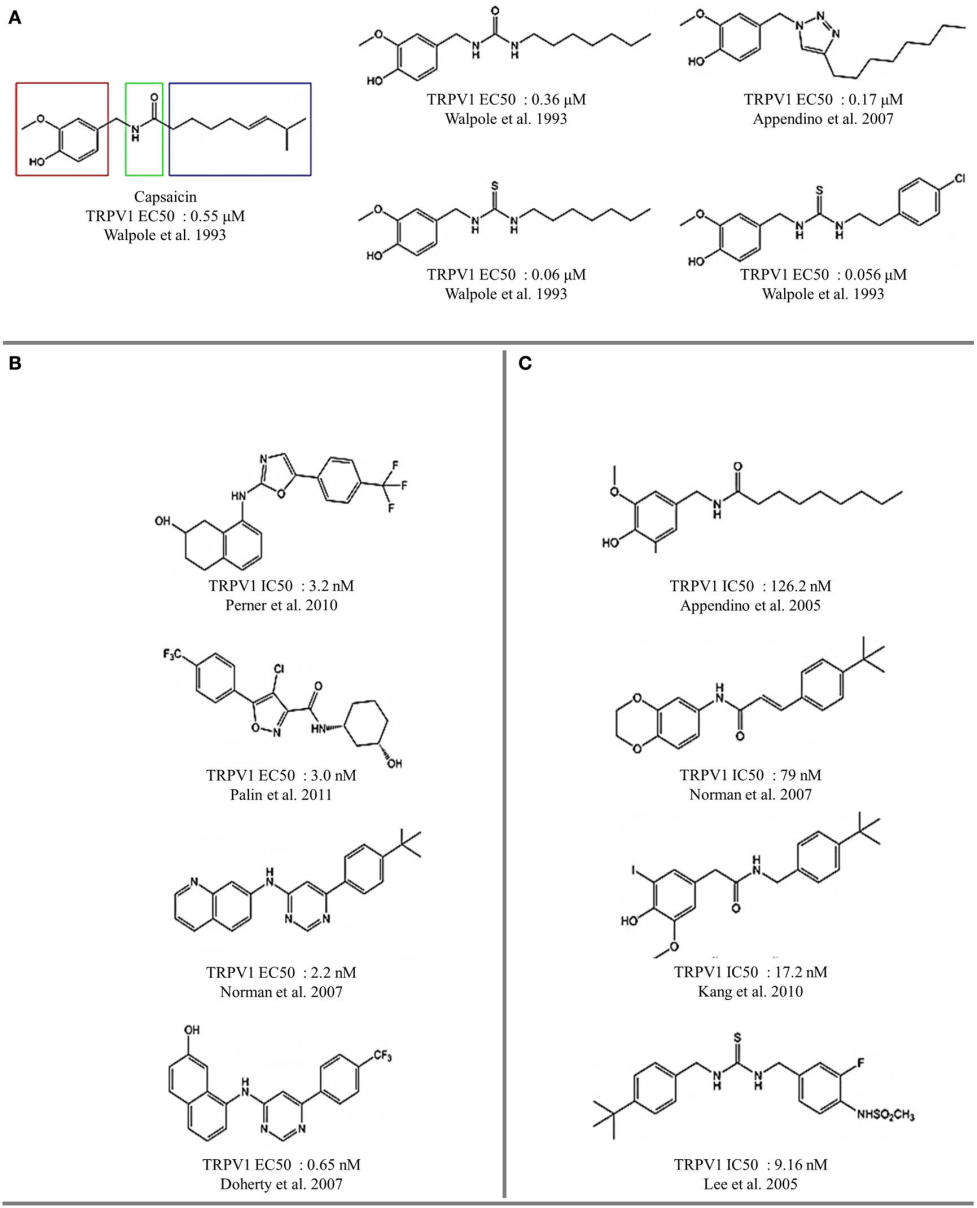
**Vanilloids and methylamines as thermoTRP channel ligands. (A)** TRPV1 agonist activity. **(B)** TRPV1 antagonist activity. **(C)** TRP channel antagonists.

Several naturally occurring compounds sharing structural similarities with capsaicin also augment the open probability of TRPV1 (Sterner and Szallasi, 1999; Vriens et al., 2008; Figures 3, 4). In this context, structure-activity relationship (SAR) analysis has proved to be a useful tool for understanding not only the effects of capsaicin's analogs, but also to find some structural requirements for competitive antagonism to inhibit the activation of TRPV1 receptors by exogenous and endogenous ligands (Walpole et al., 1993a,b,c; Planells-Cases et al., 2003; Kym et al., 2009). Capsaicin pharmacophore models (Figure 5A) consist of three parts named regions A, B, and C (Szallasi and Blumberg, 1999). In this model, recognition of the binding site is mediated by hydrogen bonds through vanillyl (region A) and amide (region B) groups. Hydrophobic interactions occur within region C through the linear aliphatic chain (3*E*)-2-methyloct-3-ene. These pharmacophoric features are frequently found in most of the identified agonists (Figure 5A) and antagonists (Figure 5B) of TRPV1 receptors. Activity measurements show that potency increases with highly polar functional groups in region B, for example urea and thiourea (Suh et al., 2005; Drizin et al., 2006). Evaluation of more restrictive conformations between regions B and C which consequently confer less flexibility to the linker connecting regions A and B (Figure 5C), enhance the antagonistic effect. Another important factor to consider is the critical role that bioactive conformations play in the design of novel drugs (Gore et al., 2007; Perner et al., 2010). The hypothesized bioactive conformations adopted for agonist and antagonist binding to TRPV1 channels show differences between the polar (B) and hydrophobic (C) regions. This observation helped the identification of several competitive antagonists, characterized by the presence of a heterocyclic system, which maintains the polar nature of region B and its ability to form hydrogen bonds (Jetter et al., 2008; Hawryluk et al., 2010).

RTX is nearly 20-fold more potent TRPV1 agonist than capsaicin. Its structure consists of a vanillyl group in region A, an ester group in region B and the tricyclic system tetradecan (known as *daphnane* ring) in the hydrophobic region. The difference in specificity with respect to capsaicin is associated with the functional group alfa, beta-unsaturated alfa-hidroxyketone in the pentameric ring. The absence of this group drives loss of activity, and modifications by 3β, 4β-diol cause a 50-fold decrease in potency (Walpole et al., 1996). Thus, the modifications of RTX suggest that the design of capsaicin-inspired pharmacophores can be extended, making identification and design of bioactive ligand conformations possible. This can be achieved through modification of hydrogen bonding and polar interactions, improving both potency and specificity.

The lipophilicity of substituent groups affects the ligand-receptor interaction hydrophobic contacts are critical for TRPV1 activation, which is in good correlation with the hydrophobic pocket present in the TRPV1 structure. Interestingly, the position of substituents influences the bioactive conformation. While the *para*- position of the ligand modulate the agonist or antagonist effect, the *meta*- position of the ligand modulate the intensity of the response. All these observations suggest that the ligand-binding sites of TRPV1 possess a high degree of plasticity, allowing for the action of different ligands through different sets of interactions within the same region.

Menthol [(1R,2S,5R)-2-isopropyl-5-methylcyclohexanol], a major component of mint (Figure 4E), possesses known cooling properties due to the activation of its pharmacological target, the cold receptor TRPM8. Interestingly, menthol affects other TRP channels, acting as TRPV3 agonist, and having a dual effect on TRPV1 and TRPA1 (Macpherson et al., 2005; Karashima et al., 2007; Xiao et al., 2008; Willis et al., 2011). Moreover, para-menthane-based TRPM8 ligands such as methylamine derivatives display good structural pharmacophore overlap with TRPV1 ligands (Ortar et al., 2010). A good example is BCTC [4-(3-chloro-pyridin-2-yl)-piperazine-1-carboxylic acid (4-tert-butyl-phenyl)-amide], a potent TRPV1 and TRPM8 antagonist. SAR analysis for BCTC analogs showed that substitution of the amide group with an ester group reduces the activity of the TRPV1 receptor, while increasing the antagonism of TRPA1 (Morera et al., 2009). Usually non-para-menthane based ligands have an antagonistic effect on TRPM8 channels. One important observation is that the super-cooling agent icilin, is the only non-para-menthane based TRPM8 ligand acting as an agonist (Journigan and Zaveri, 2013). Notably icilin, initially synthesized as a central nervous system depressant (Do and Podesva, 1974), has nearly 200-fold greater efficacy than menthol (McKemy et al., 2002). As for the case of menthol, icilin also has a dual action on TRPA1 channels (Story et al., 2003).

## Generalizing the action of PUFAs over hot and cold receptors

**Figure 6:**
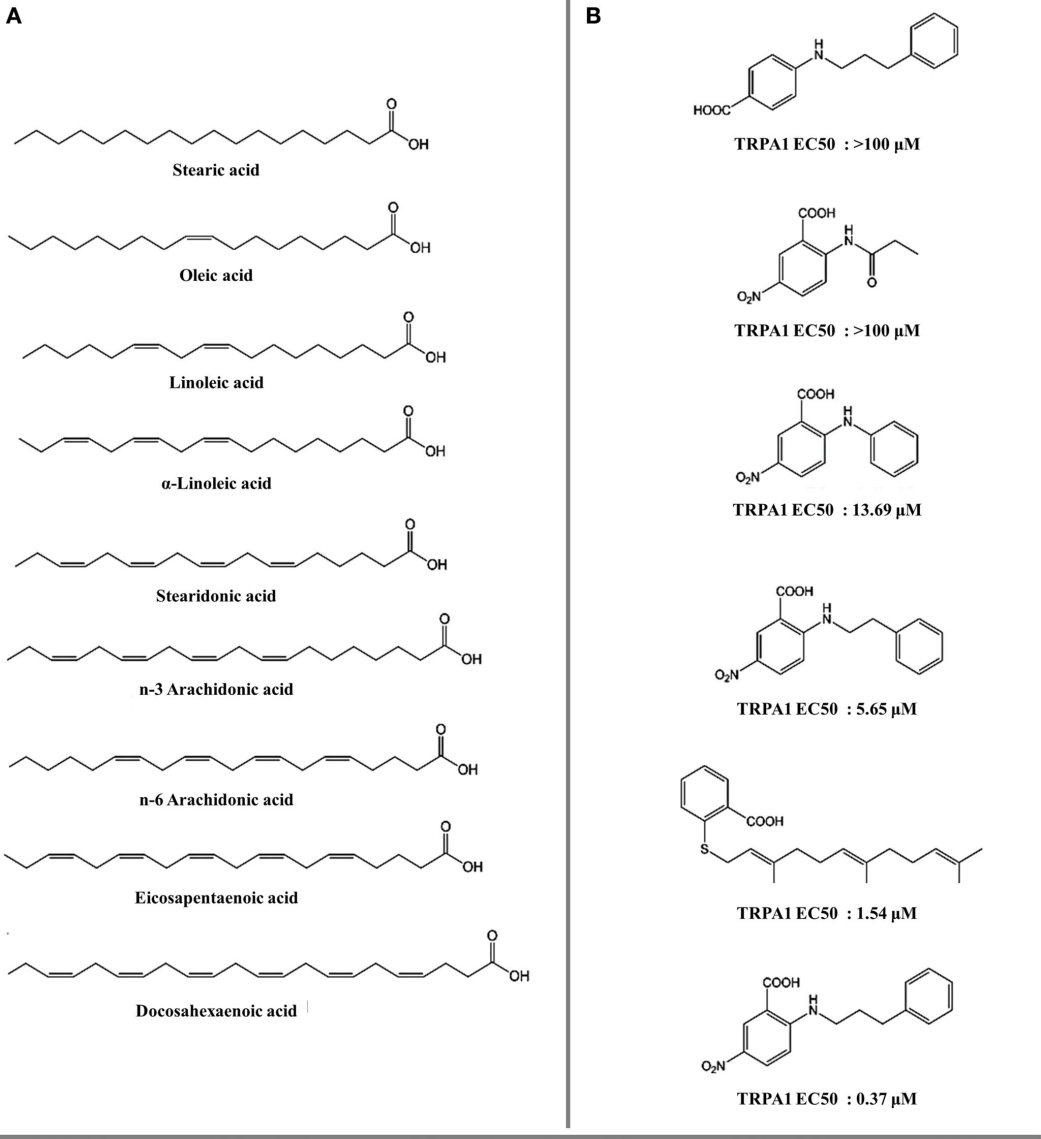
**Fatty acids and lipids as thermoTRP channel ligands. (A)** Polyunsaturated fatty acids (PUFAs) evaluated on TRPV1 and TRPA1 receptors (Matta et al., 2007; Motter and Ahern, 2012). **(B)** 5-nitro-2-(3-phenylpropylamino) benzoic acid derivatives evaluated on TRPA1 receptors (Liu et al., 2010).

Omega-3 polyunsaturated fatty acids (PUFAs) and some of their derivatives, for example farnesyl thiosalicylic acid (Maher et al., 2008) and 5-nitro-2-(3-phenylpropylamino) benzoic acid (NPPB) (Liu et al., 2010) are moderate agonists of TRPV1 (Matta et al., 2007) and TRPA1 (Motter and Ahern, 2012). Lipids bind differently on TRPV1 compared with capsaicin (Figure 1C). SAR analysis of PUFAs indicates that the aromatic ring is critical for their action. On the other hand, increasing unsaturation of the fatty acid polar domain promotes a more potent response (Figures 6A,B).

The inherent promiscuity of thermoTRP's is evident in the case of leucettamols, a class of marine sphingolipids. While the majority of leucettamols act as TRPM8 inhibitors and TRPA1 activators, only leucettamol B acts as a TRPV1 agonist (Chianese et al., 2012) (Figure 4A). Interestingly, both PUFAs and leucettamols display an unsaturated system, however PUFAs possess a carboxylic acid at the polar region, while leucettamols contain two amine and two hydroxyl groups (Figure 4A). Since PUFAs are general TRPV1 activators, it has been suggested that agonist activity toward TRPV1 is mainly influenced by a polar component that favors ligand recognition, leading to channel opening. An equivalent mechanism could be envisioned for the antagonistic effect observed in TRPM8 receptors, which will be leaded by a less polar component of the leucettamol molecule.

## Concluding remarks

It has been reported that an extensive number of natural compounds of aliphatic nature either endogenous or exogenous, and synthetic compounds modulate thermoTRP channel activity. In the present review we illustrate that thermoTRP channel ligand-binding sites engage different electrostatic, hydrophobic and steric factors, while retaining a set of conserved structural features shared by all members. General rules can be drawn for ligand stabilization to the bioactive conformation which seems to be highly promoted by the existence of polar contacts and rigidness of the molecule, primarily affecting agonist activity and potency responses. Weaker interactions such as hydrogen bonds are likely involved in defining ligand response as agonistic or antagonistic. The recently released structural data of TRPV1 channels will allow for the exploration of all these differences. Molecular docking studies together with site-directed mutagenesis are likely to be useful in helping to solve these questions; computational methods are emerging as powerful tools which have the potential to support iterative cycles of theoretical prediction, experimental testing and further refinement to achieve greater understanding.

### Conflict of interest statement

The authors declare that the research was conducted in the absence of any commercial or financial relationships that could be construed as a potential conflict of interest.

## Footnotes

^1^Note: Kir Channels posses only two transmembrane helices the outer, similar to TM5, and the inner helix, akin to TM6. These helices are associated to the intracellular domains of the channel by helical linkers. While the *tether helix* connects the inner helix with the cytoplasmatic C-terminal, the *interfacial (IF) helix* connects the outer helix with the cytoplasmatic N-terminal.

## Abbreviations

ARD: ankiryn repeat domain
BCTC: 4-(3-chloro-pyridin-2-yl)-piperazine-1-carboxylic acid (4-tert-butyl- phenyl)-amide
CaV: Voltage-gated calcium cannel
CNG: cyclic nucleotide-gated cannel
CPZ: Capsazepin
Cryo-EM: Cryo-electron microscopy
DkTx: double-knot vanollotoxin
G-V: Conductance-Voltage curve
HCN: Hyperpolarization-activated and cyclic nucleotide-gated cannel
Kir: inwardly rectifying potassium cannel
Kv: Voltage-gated potassium cannel
LPA: lysophosphatidic acid
MD: molecular dynamics
Menthol: 1R,2S,5R-2-isopropyl-5-methylcyclohexanol
NADA: N-arachidonoyl-dopamine
NPPB: 5-nitro-2-(3-phenylpropylamino) benzoic acid
PIP2: phosphatidylinositol biphosphate
PI(4,5)P2: phosphatidylinositol 4,5-biphosphate
PUFA: polyunsaturated fatty acids
RTX: resiniferatoxin
SAR analysis: Structure-activity relationship analysis
SCAM: substituted cysteine accessibility method
TD: TRP-domain
TM: transmembrane-spanning hélix
TRP: Transient Receptor Potential
VSD: Voltage-sensing Domain.
